# The relationship between psychological readiness to return to sport and kinesiophobia in teens and young adults after anterior cruciate ligament reconstruction

**DOI:** 10.3389/fpsyg.2025.1623398

**Published:** 2025-10-10

**Authors:** Lauren Butler, Shelby Baez, Cody Walker, Dylan Roman, Taylor Douthit, Christopher Kuenze, Sophia Ulman, ARROW

**Affiliations:** ^1^Department of Physical Therapy, Florida International University, Miami, FL, United States; ^2^Department of Rehabilitation, Nicklaus Children’s Hospital, Miami, FL, United States; ^3^Department of Exercise and Sport Science, University of North Carolina at Chapel Hill, Chapel Hill, NC, United States; ^4^Department of Orthopedic and Sports Medicine, Arkansas Children’s Hospital, Little Rock, AR, United States; ^5^Department of Sports Physical Therapy, Connecticut Children’s, Hartford, CT, United States; ^6^Department of Sports Rehabilitation, Children’s Health Andrews Institute, Plano, TX, United States; ^7^Department of Orthopaedic Surgery, University of Texas Southwestern Medical Center, Dallas, TX, United States; ^8^Department of Kinesiology, University of Virginia, Charlottesville, VA, United States; ^9^Movement Science Lab, Scottish Rite for Children, Frisco, TX, United States

**Keywords:** ACL reconstruction, psychology, patient-reported outcomes, return to sport, fear of re-injury

## Abstract

**Introduction:**

Psychological readiness and kinesiophobia are important variables to consider for return to sport clearance after anterior cruciate ligament (ACL) reconstruction. Both have been associated in adult populations; however, it is unknown if they are associated in teens after ACL reconstruction. Therefore, the purpose of this study was to assess the relationship between psychological readiness and kinesiophobia in teens and young adults after ACL reconstruction.

**Methods:**

A retrospective cohort design was used. Participants aged 13–30 years, who were 6–12 months post-ACL reconstruction, who completed the Tampa Scale of Kinesiophobia and the ACL Return-to-Sport after Injury Scale were included from a multi-site registry. Two age groups were established (teen: <19 years, adult: ≥19 years), and psychological readiness was categorized using an ACL Return-to-Sport after Injury Scale cutoff of 77 (<77 = unacceptable). Independent samples *t*-tests, Pearson correlations, and binary logistic regression were performed to examine associations between kinesiophobia and psychological readiness, and the influences of age, sex, and months since surgery.

**Results:**

315 participants (54.3% female; 18.3 ± 3.3 years; 8.2 ± 1.9 months post-surgery) were analyzed. ACL Return-to-Sport after Injury Scale and Tampa Scale of Kinesiophobia scores were significantly correlated in both groups (teen: *r* = −0.59, *p* < 0.001; adult: *r* = −0.45, *p* < 0.001), with no significant difference in the correlation coefficients (z = −1.49). Overall, 47.9% scored below the ACL Return-to-Sport after Injury pass threshold. Each one-point increase in kinesiophobia was associated with a 28% higher likelihood of reporting unacceptable psychological readiness. Adults were twice as likely as teens to report unacceptable psychological readiness.

**Discussion:**

Greater psychological readiness was associated with lower kinesiophobia in both teens and young adults. Additionally, nearly half reported poor psychological readiness, highlighting the need for interventions aimed at improving psychological readiness during ACL rehabilitation.

## 1 Introduction

Anterior cruciate ligament (ACL) injuries can significantly impact a young athlete’s (under 30 years of age) career. Sustaining an ACL tear typically results in surgical reconstruction followed by 6–12 months of rehabilitation with only roughly half of athletes making a full return to sport within 1 year after surgery (Ardern et al., 2011). Given the low rates of return to sport following primary ACL reconstruction (ACLR), researchers and clinicians have sought to identify factors associated with poor outcomes in athletes who have undergone primary ACLR.

Late-stage rehabilitation and the process of returning to sport can be mentally challenging for athletes who have had an ACLR (DiSanti et al., 2018). Negative emotions such as fear, anxiety, and decreased confidence may resurface as the athlete is preparing to return to sport (RTS) (Johnston and Carroll, 1998; Morrey et al., 1999; Kvist et al., 2005; Clement et al., 2015; Sonesson et al., 2017). An athlete’s psychological readiness to RTS has been identified as an important factor to consider after ACLR (Ardern et al., 2013; Ardern et al., 2014; Ardern et al., 2016; Forsdyke et al., 2016). A systematic review of psychological factors associated with outcomes after sport related injury found that restoring an athlete’s self-confidence was a key theme to achieving positive RTS outcomes (Forsdyke et al., 2016). The authors suggested that confidence, both in the injured body part and in the ability to perform, may act as a buffer for injury-related fear and ultimately allow the athlete to achieve mental readiness to RTS (Garnefski et al., 2002; Forsdyke et al., 2016). In the context of ACLR, it has been well documented that an athlete’s psychological readiness is associated with their ability to RTS (Ardern et al., 2014; Czuppon et al., 2014; Fones et al., 2020; Welling et al., 2020). Age and sex appear to influence this construct after ACLR with younger patients (less than 18 years of age) and males reporting greater readiness compared to older patients and females (Webster et al., 2018; Kostyun et al., 2021; Milewski et al., 2023). Moreover, lower psychological readiness at the time of RTS has been associated with a higher risk of recurrent ACL injuries in athletes aged 20 years and younger, while no such relationship has been reported in athletes over the age of 20 (McPherson et al., 2019). These age-related differences may be influenced by developmental or experience related psychological factors that affect risk appraisal (Garnefski et al., 2002; Silvers et al., 2012; Deng et al., 2019).

Kinesiophobia, often described as pain-related fear, fear of movement, and/or fear of reinjury, has also been identified as a barrier to RTS and a predictor of poor outcomes after ACLR in youth athletes (Lundberg et al., 2011; DiSanti et al., 2018). A qualitative study of youth athletes after ACLR who had not yet been cleared to RTS reported consistent psychosocial barriers associated with RTS including the perception that sport activities were now linked to injury (DiSanti et al., 2018). Higher kinesiophobia has also been associated with decreased hop performance, reduced quadriceps strength, and increased risk of second injury in youth athletes after ACLR (Paterno et al., 2018). Specifically, Paterno et al. (2018) reported that patients with higher kinesiophobia greater than or equal to 17 on the Tampa Scale of Kinesiophobia (TSK-11) are four times more likely to report lower physical activity levels (odds ratio 3.73; 95% CI, 0.98–14.23), seven times more likely to demonstrate hop test asymmetry (odds ratio 7.1; 95% CI, 1.5–33.0), and six times more likely to have quadriceps strength asymmetry (odds ratio 6.0; 95% CI, 1.3–27.8) (Paterno et al., 2018). The same authors reported that patients with high kinesiophobia (TSK-11 score of 19 or greater) at the time of RTS were 13 times more likely to suffer a graft rupture within 20 years of RTS compared to those with lower kinesiophobia (Paterno et al., 2018). Finally, kinesiophobia has been identified by many as a contributing factor for failure to RTS (Ardern et al., 2013; Lentz et al., 2015; Nwachukwu et al., 2019). Sex based differences in kinesiophobia after ACLR have also been reported with females citing fear of re-injury as a reason for not returning to their prior level of sport participation more frequently than males (Kvist et al., 2005). It has been suggested that differences in perceptions of re-injury related risk between males and females may be an influential factor on RTS decision making and may be one reason why females report lower RTS rates than males (Siegel, 2024).

Given the impact of psychological constructs on RTS after ACLR, assessment of these factors has become an important component of RTS decision making. The Anterior Cruciate Ligament–Return to Sport After Injury Scale (ACL-RSI) and the TSK-11 are commonly used to assess psychological readiness and kinesiophobia after ACLR, respectively (Woby et al., 2005; Webster et al., 2008; Webster and Feller, 2018; Cirrincione et al., 2023). Although psychological readiness and kinesiophobia are two unique constructs, several papers have reported correlations between ACL-RSI and TSK-11 scores in the adult population after ACLR (Kvist et al., 2013; Harput et al., 2017; Sala-Barat et al., 2020). Significant negative correlations have been reported between the ACL-RSI and the TSK-11, ranging from weak to strong, suggesting that greater psychological readiness to RTS may be associated with lower kinesiophobia (Kvist et al., 2013; Harput et al., 2017; Slagers et al., 2017; Sala-Barat et al., 2020). Understanding this association is important to guide interventions that target psychological readiness and kinesiophobia during rehabilitation after ACLR, as strategies to reduce kinesiophobia may differ from those focused on addressing emotions and confidence. However, we do not know whether these correlations exist in teens (13–18 chronological years of age). Psychological changes related to growth and development should be considered when assessing these constructs for RTS decision-making in teens. Additionally, time from surgery is another important factor influencing psychological outcomes after ACLR, with greater mental readiness to RTS observed at later post-operative time points (Kostyun et al., 2021). Given that youth athletes are at the highest risk of ACL injury recurrence, and with the rising concerns related to athlete mental health,(Daley and Reardon, 2024) it is imperative that we gain a deeper understanding of the relationship between these two psychological constructs in this population.

Thus, the purpose of this study was to assess the relationship between psychological readiness and kinesiophobia in teens (13–18 years of age) and young adults (19–30 years of age) after primary ACLR. Our hypothesis was that there would be an inverse relationship between psychological readiness and kinesiophobia after ACLR, and that the strength of the relationship would vary by age and sex.

## 2 Methods

This was a multisite study including teen and young adult participants from two sites, one large pediatric hospital and one university-based sports medicine clinic, included in the ACL Reconstruction Rehabilitation Outcomes Workgroup (ARROW) clinical outcomes registry. The purpose of ARROW is to combine data and resources from a geographically diverse consortium of researchers at affiliated universities, hospitals, and research sites to improve clinical decision-making and patient care following ACLR. Data were collected as a part of separate Institutional Review Board (IRB) approved research studies at each site, then a limited data set from each site was aggregated in an IRB approved registry housed and managed by study team members at the University of Virginia (HSR 230335).

### 2.1 Participants

Participants were included if they underwent primary ACLR between 2013 and 2021, were between 13 and 30 years of age, and completed the ACL-RSI and the TSK-11 between 6 and 12 months after primary ACLR. Participants were excluded if they were missing either questionnaire or had a history of contralateral or ipsilateral ACLR. Participants at all sites completed the ACL-RSI and the TSK-11 as part of a battery of functional tests, including assessments of muscular strength and hop test performance, between 6 to 12 months from primary ACLR surgery. If the battery of functional tests was completed more than once between the 6- and 12-month period, the test closest to 12 months was used to best match with RTS. Demographic and surgical information including age, sex, and time from surgery to RTS test were also collected. Rehabilitation protocols used at each site were summarized in Appendices A, B.

### 2.2 Outcome measures

The ACL-RSI is a 12-item scale that measures psychological readiness to RTS after ACLR. The scale assesses three constructs: emotional response, confidence in performance, and risk appraisal. The ACL-RSI is scored from 0 to 100, with higher scores indicating greater psychological readiness. The ACL-RSI has demonstrated high validity and internal consistency (Cronbach’s *α* = 0.96) (Webster et al., 2018). Due to registry limitations and a lack of available data for the ACL-RSI subscales, the total score was used in this study.

The TSK-11 is an 11-item questionnaire designed to assess kinesiophobia, with scores ranging from11 to 44 (minimal kinesiophobia to highest kinesiophobia). The TSK-11 has been validated for use in patients after ACLR (George et al., 2012). The tool has demonstrated excellent internal consistency (Cronbach’s α = 0.79) and good reliability (intraclass correlation coefficient = 0.81) (Woby et al., 2005).

### 2.3 Statistical analysis

Means and standard deviations were computed for all continuous variables, including age, months since surgery, ACL-RSI total score, and TSK-11 total score. Two age groups were established with the ‘teen’ group including participants less than 19 years of age and the ‘young adult’ group including participants at or above 19 years of age. These age groups were selected based on the work of Butler et al., who established age categories in sports medicine research based on chronological age: early teen (ages 13–15 years), late teen (ages 16–18 years), and adults (age 19 years and older) (Butler et al., 2023). Since the younger age group in this study spans both early and late teens, we have defined this group collectively as ‘teens’. Additionally, two subgroups were established based on ‘acceptable’ and ‘unacceptable’ ACL-RSI scores using a clinical cutoff of 77 (McPherson et al., 2019). This cutoff was chosen by the work of Mcpherson et al. who reported that a score of 77 points on the ACL-RSI demonstrated 90% sensitivity in identifying those who went on to suffer a second ACL injury (McPherson et al., 2019). Thus, participants that scored less than the pass threshold of 77 were determined to report an unacceptable ACL-RSI score, indicating poor psychological readiness. Independent samples *t*-tests were performed to identify significant sex and age group differences in TSK-11 and ACL-RSI total scores and a significant difference in TSK-11 by ACL-RSI subgroups. Cohen’s *d* effect sizes were calculated to quantify the magnitude of these group differences (Cohen, 1988) significance level (*α*) was set to 0.05.

Pearson correlations were performed to determine significant associations between ACL-RSI and TSK-11 total scores within each age group, and Fisher’s Z transformation was conducted to statistically test whether the correlation coefficients differed between the two age groups. Additionally, Pearson correlations were performed to determine significant associations between months since surgery and both ACL-RSI and TSK-11 total scores. A Bonferroni correction was applied to account for multiple comparisons across the five correlations, adjusting the significance threshold to *p* < 0.010. Correlations were identified as weak (*r* ≤ 0.35), moderate (*r* = 0.36–0.67), and strong (*r* ≥ 0.68) (Taylor, 1990). Subsequently, a binary logistic regression was performed to determine the association between TSK-11 total score and the likelihood of reporting an unacceptable ACL-RSI score. Biological sex, teen vs. young adult group, and months since surgery were included in the regression model as covariates. Specifically, months since surgery was included as greater mental readiness to RTS has been observed at later post-operative time points (Kostyun et al., 2021). Odds ratios and their 95% confidence intervals were extracted from the regression output to quantify the strength and precision of the associations. All analyses were performed using SPSS Statistics (IBM SPSS Statistics for Windows, version 224.0, Armonk, NY, USA).

## 3 Results

**Figure 1:**
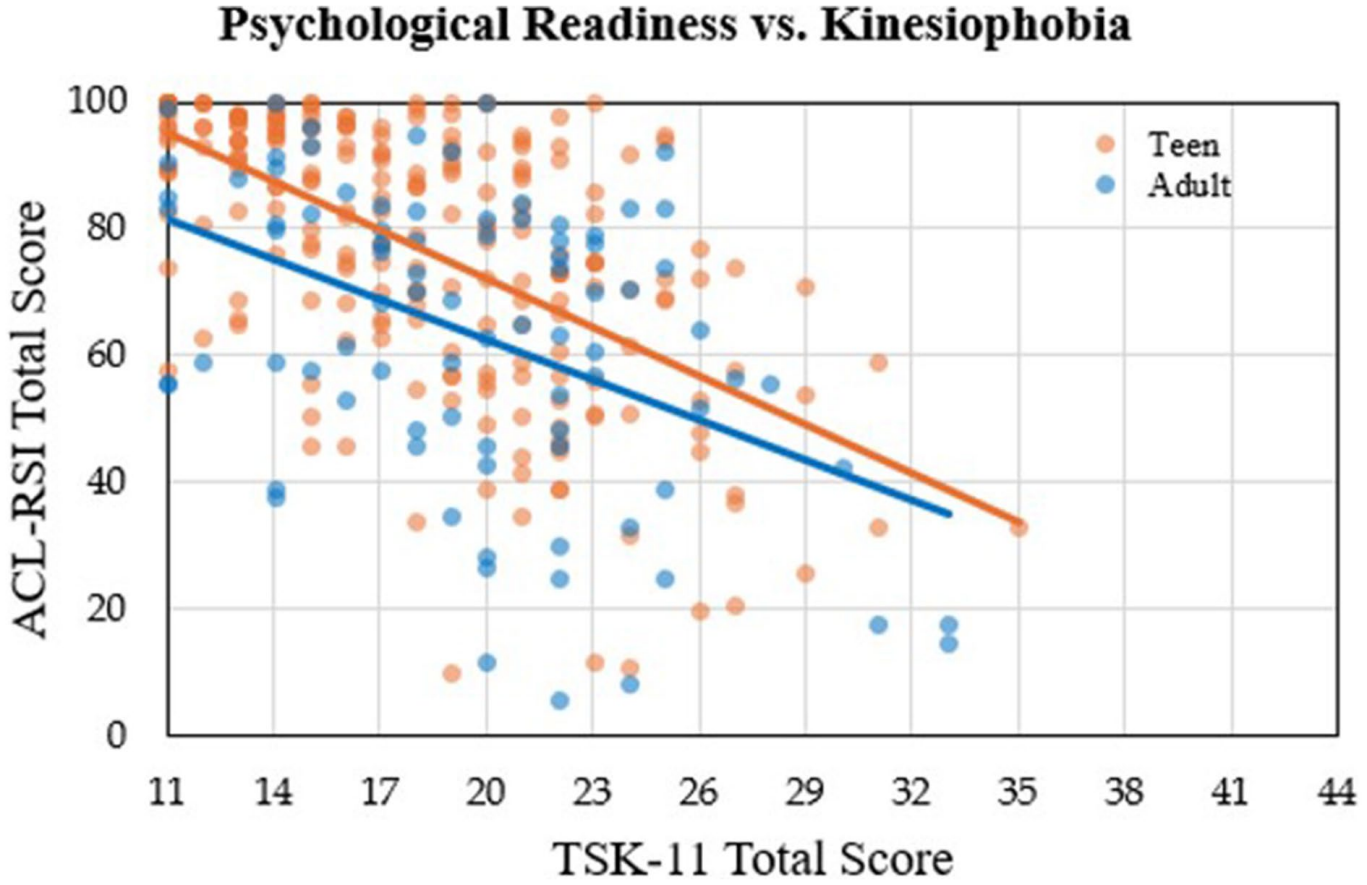
Psychological readiness (ACL-RSI total score) versus kinesiophobia (TSK-11 total score) depicted for both age groups. The trendline for the teen group is orange and the trendline for the adult group is blue.

A total of 315 participants (54.3% female; 72.7% teens; 18.3 ± 3.3 years; 8.2 ± 1.9 months from surgery) were included for analysis (Table 1). No sex differences were found in ACL-RSI or TSK-11 scores regardless of age. Alternatively, significant age group differences were found in both ACL-RSI (mean difference: 14.0, *p* < 0.001, *d* = 0.64) and TSK-11 (mean difference: 1.6, *p =* 0.007, *d* = 0.32), with teens reporting overall greater psychological readiness and reduced levels of kinesiophobia. A moderate inverse correlation was observed between ACL-RSI and TSK-11 scores (*r* = −0.56, *p* < 0.001) such that greater psychological readiness was associated with lower levels of kinesiophobia. Similarly, within each age group, ACL-RSI and TSK-11 total scores were significantly correlated (teen: *r* = −0.59, *p* = < 0.001; adult: *r* = −0.45, *p* = < 0.001), and the correlation coefficients for each age group did not significantly differ (*z* = −1.49) (Figure 1). An increase in months since surgery was weakly correlated with greater psychological readiness (*r* = 0.17, *p* = 0.003).

**Table 1:** Demographics, ACL-RSI score, and TSK-11 score by age group and sex.

Variable	Teen	Adult	*p*	Male	Female	*p*	Total
% cohort	72.7%	27.4%	–	45.9%	54.3%	–	–
Age (years)	16.7 ± 1.3	22.6 ± 2.9*	<0.001	18.8 ± 3.7	17.9 ± 2.8*	0.007	18.3 ± 3.3
MSS	8.2 ± 1.8	8.4 ± 1.9	0.142	8.2 ± 2.0	8.3 ± 1.8	0.287	8.2 ± 1.9
ACL-RSI Score	77.2 ± 21.1	63.3 ± 23.8*	<0.001	72.9 ± 22.9	73.8 ± 22.6	0.361	73.4 ± 22.7
TSK-11 Score	18.1 ± 4.8	19.6 ± 5.0*	0.007	18.9 ± 5.2	18.2 ± 4.7	0.100	18.5 ± 4.9

Significant group differences are noted with an asterisk. MSS, months since surgery.

Across the cohort, the total ACL-RSI and TSK-11 scores averaged 73.42 ± 22.68 and 18.50 ± 4.92, respectively. Overall, 47.9% of participants scored below the ACL-RSI pass threshold (<77), indicating poor psychological readiness. Participants with poor psychological readiness reported significantly greater kinesiophobia (20.97 ± 4.72) as compared to participants with acceptable psychological readiness (16.14 ± 3.92, *p* < 0.001, *d* = 2.81). Similarly, the logistic regression indicated that a greater kinesiophobia was associated with an unacceptable psychological readiness (*Χ*^2^(4) = 89.87, *p* < 0.001; Table 2). For every one-point greater increase in TSK-11 score there was a 28% increase in the odds of having an ACL-RSI score below the pass threshold, when controlling for age, sex and months since surgery. Additionally, adults were twice as likely to report an unacceptable ACL-RSI score. Sex and time since surgery were not found to be significant covariates in the regression model.

**Table 2:** Regression findings to determine the association between unacceptable ACL-RSI scores and TSK-11 score.

Variable	Estimate	Std. Error	*p*-value	Odds Ratio	Lower 95% CI	Upper 95% CI
Intercept	−4.10	0.88	<0.001*	0.02	–	–
Sex	0.32	0.27	0.233	1.38	0.81	2.32
Age Group	0.69	0.30	0.021*	2.00	1.11	3.61
MSS	−0.11	0.07	0.138	0.90	0.78	1.04
TSK-11	0.25	0.03	<0.001*	1.28	1.20	1.36

*Significance (α = 0.05). MSS, months since surgery.

## 4 Discussion

The purpose of this study was to assess the relationship between psychological readiness and kinesiophobia in teens and young adults 6–12 months after primary ACLR. Our hypothesis, that there would be an inverse relationship between psychological readiness and kinesiophobia, was supported. A moderate negative correlation was observed between ACL-RSI and TSK-11 scores in both teens and young adults, indicating that greater psychological readiness was associated with lower levels of kinesiophobia. However, there was no difference in the strength of the correlation between teens and young adults. Across the cohort, greater TSK-11 scores were associated with unacceptable ACL-RSI scores, with 28% increased odds of having an unacceptable ACL-RSI score for every one-point increase in TSK-11 score.

These findings align with previous studies in adult populations aged 16 to 40 years, which have reported associations between psychological readiness and kinesiophobia (Sheridan et al., 2014; Lentz et al., 2015; Sonesson et al., 2017; Isaji et al., 2024). Despite differences in psychological and emotional responses between teens and adults, in our participants, psychological readiness and kinesiophobia maintained a relationship across different age groups. While a relationship was found in this study, the significant contribution of covariates in improving the overall model suggests that while broad measures of psychological response after ACLR are helpful as part of clinical decision making, without evaluation of specific constructs, implementing effective interventions may be challenging. The ACL-RSI contains three subscales: emotion, confidence, and risk appraisal. While this study only used the total score, which is often utilized in clinical care, future research may benefit from examining subscale scores which may help reveal deficiencies in areas that targeted interventions would be useful (Aizawa et al., 2024). Furthermore, athletes often have an emotional response to injuries that limit their participation in sport. This is heightened during the return to sport phase, further supporting the need to identify what areas of the subscale interventions should be targeted to (Webster et al., 2008). Given this and the relationship between the ACL-RSI and the TSK-11, future studies should also examine whether interventions aimed to address negative emotions and increase confidence after ACLR have an effect on athletes’ kinesiophobia.

Overall, nearly half (48%) of participants indicated unacceptable ACL-RSI scores based on the cutoff of 77 reported by McPherson et al. with a mean ACL-RSI score of 73.4 (McPherson et al., 2019). This finding suggests that at approximately 8 months after ACLR, these athletes were not psychologically ready to RTS. This trend is corroborated by the work of Thorolfsson et al. (2023) who reported a mean ACL-RSI score of 71 in a group of 347 adolescents with a mean age of 17.1 years at a similar post-operative timepoint (Thorolfsson et al., 2023). Similarly, in a slightly older group of athletes (mean age 28 ± 10 years), Webster et al. reported mean ACL-RSI scores of 65 at an average of 12 months after primary ACLR (Webster et al., 2018). These findings support the need for targeted interventions, such as video modeling of athletes performing skills in the different stages of rehabilitation and eventually RTS or guided imagery techniques, (Coronado et al., 2018; Isaji et al., 2024) during ACLR rehabilitation to improve athletes’ psychological readiness to return to sport.

This study also found that young adult participants were twice as likely to report an unacceptable ACL-RSI score compared to teen participants (OR = 2.00, 95% CI [1.11, 3.61]). This is supported by other works that have found age-related differences in psychological readiness to RTS after ACLR with younger patients reporting greater readiness (Webster et al., 2018; Kostyun et al., 2021; Milewski et al., 2023). Psychological and emotional responses to stressors differ between adults and adolescents which may have contributed to these findings (Garnefski et al., 2002; Silvers et al., 2012; Deng et al., 2019). Compared to adults, adolescents’ cognitive appraisal of a situation is more affected by their current emotional state rather than through engaging in effective decision-making strategies (Garnefski et al., 2002; Silvers et al., 2012; Deng et al., 2019). It is plausible that despite consistent feelings of injury related fear associated with RTS, perceived psychological readiness may remain high. This may be due to the altered cognitive appraisal of the risk associated with returning to sport. Furthermore, teens may have greater motivation to RTS, as sports participation offers a means of social engagement that may no longer be available to young adults who have moved beyond their youth sports experiences (Cirrincione et al., 2023). This has been highlighted in prior works which have reported an interaction between the social environment and an athlete’s motivation (Podlog and Eklund, 2006; Hildingsson et al., 2018). This emotional desire to regain social connection may supersede injury related fear and alter the athletes’ risk appraisal of returning to sport (Gross and John, 2003; Sheridan et al., 2014).

Importantly, the mean age of the teen group in this study was 16.7 ± 1.3 years representing a “late teen” group of athletes (Butler et al., 2023). Age-related differences in the association between psychological readiness and kinesiophobia may be more apparent in children (5–12 years of age) and early teen (13–15 years of age) athletes (Christie and Viner, 2005). The average age of pubertal onset is reported at 10.8 years in females versus 12.9 years in males (Aris et al., 2022). It is possible that the majority of the athletes in this study may have already gone through puberty. Thus, their emotional maturity may be more developed and approaching that of adults. Future studies should explore the association between psychological readiness and kinesiophobia in athletes under 13 years old to gain a deeper understanding of how age influences this association. Future studies should also aim to validate the ACL-RSI and the TSK-11 in this age group.

Finally, no sex-based differences in the relationship between the ACL-RSI and the TSK-11 were observed. While sex-based differences in psychological response to injury have been reported, these findings suggest that psychological readiness to RTS and kinesiophobia maintained a relationship across sexes. However, given that sex-based differences in ACL-RSI subscale scores have been reported, future work should explore the relationship between kinesiophobia and each subscale to better understand the relationship (Webster and Feller, 2022).

### 4.1 Limitations

This study has several limitations that need to be discussed. First, due to the older age of the teen group in this study, our findings may not be generalizable to a younger athlete population and thus future studies should repeat similar investigations with early teens and children. Next, this was a multi-site study which presented difficulties in controlling rehabilitation protocols used across the different treatment centers. However, the outcomes of psychological readiness and kinesiophobia (ACL-RSI and TSK-11) analyzed in the current study were captured via standardized questionnaires and thus consistent across sites. This study did not consider concomitant surgical procedures and other surgical characteristics. Due to limitations in the registry, we were not able to ascertain whether the athletes actually returned to their sport. This is a key limitation given that both psychological readiness and kinesiophobia are most relevant in the context of RTS. Additionally, we did not capture information about prior sports participation or the desire to RTS, which may have influenced study findings. Lastly, due to registry limitations, the study team only had access to the total ACL-RSI scores. Future studies should examine subscales of the ACL-RSI to assess the relationship between sex and age with the emotions, confidence, and risk appraisal subscales. The variation in rehabilitation approaches, the lack of concomitant surgical data, and the absence of actual RTS outcomes may have influenced psychological responses and thus weaken the interpretation of study findings.

## 5 Conclusion

This study found that greater psychological readiness was associated with lower kinesiophobia after ACLR regardless of age or sex. For every one-point increase in TSK-11 score, there was 28% increased odds of having an unacceptable ACL-RSI score. Overall, 48% of participants reported unacceptable psychological readiness to RTS, with a higher likelihood of having an unacceptable ACL-RSI score in older participants compared to younger participants. Based on literature and the results presented in the current study, health care providers may not adequately be addressing psychological constructs related to readiness to RTS in patients after ACLR. This highlights the importance of including interventions aimed at improving psychological readiness throughout the rehabilitation process, with extra attention on older athletes. Given the relationship between psychological readiness and kinesiophobia, future studies should examine if interventions aimed to reduce kinesiophobia, such as guided imagery, positive self-talk, and graded exposure, have a positive effect on psychological readiness to RTS after ACLR.

## Data Availability

The original contributions presented in the study are included in the article/Supplementary material, further inquiries can be directed to the corresponding author.
