# Supplementary material for: The relationship between psychological readiness to return to sport and kinesiophobia in teens and young adults after anterior cruciate ligament reconstruction
Source: Front Psychol. 2025 Oct 10;16:1623398. doi: 10.3389/fpsyg.2025.1623398 (PMC12549314; doi:10.3389/fpsyg.2025.1623398)
Supplement: Supplementary file 1 [file Data_Sheet_1.docx]

**Appendix A**

***Site 1 ACL Reconstruction Rehabilitation Protocol***

Treatment: See attached ACL Rehabilitation Protocol

Weight bearing status: WBAT (TDWB x 6 weeks if meniscal repair)

Brace:

Frequency/Duration: 2-3x/week x 12 weeks

First six weeks post-op:

ACL hinged knee brace (TROM or equivalent) for weight bearing activities:

May start w/ locked, but unlock as soon as quad control returns (3 - 5 days postop)

Brace range: 0 to 90 degrees

Brace locked at 0 degrees for sleep

**Weeks 6 to 14:**

BTB/Quad Tendon: change to simple hinge at 6 weeks

Hamstring: change to simple hinge at 12 weeks

No scar massage until 6 weeks post-op

No PROM in flexion.

*Phase 0:* Pre-operative Recommendations

Normal gait

AROM 0 to 120 degrees of flexion Strength: 20 SLR with no lag Minimal effusion

Patient education on post-operative exercises and need for compliance

Educated in ambulation with crutches

Wound care instructions

*Phase 1:* Immediate Post-operative Phase

(Approximate time frame: surgery to 2 weeks)

Goals

Full knee extension ROM

Good quadriceps control (> 20 no lag SLR) Minimize pain

Minimize swelling

Normal gait pattern

Crutch Use:TDWB x 6 weeks

Brace:First six weeks, unlocked when ambulating. Lock in full extension for 5-6 hours per 24 hour period (ok during sleep)

Cryotherapy:Cold with compression/elevation (e.g. Cryo-cuff, ice with compressive stocking) First 24 hours or until acute inflammation is controlled: every hour for 15 minutes After acute inflammation is controlled: 3 times a day for 15 minutes

EXERCISE SUGGESTIONS ROM

Extension: Low load, long duration (~5 minutes) stretching (e.g., heel prop, prone hang minimizing

co-contraction and nocioceptor response)

Flexion: Wall slides, heel slides, seated assisted knee flexion, bike: rocking-for-range

Patellar mobilization (medial/lateral mobilization initially followed by superior/inferior direction while monitoring reaction to effusion and ROM)

Muscle Activation/Strength

Quadriceps sets emphasizing vastus lateralis and vastus medialis activation

SLR emphasizing no lag

Electric Stimulation: Optional if unable to perform no lag SLR Discontinue use when able to perform 20 no lag SLR

Double-leg wall slides or mini-squats without knee over foot.

Standing theraband resisted terminal knee extension (TKE) Hamstring sets

Hamstring curls

Side-lying hip adduction/abduction (Avoid adduction moment in this phase with concomitant grade II - III MCL

injury)

Quad/ham co-contraction supine

Prone Hip Extension

Ankle pumps with theraband

Heel raises (calf press)

Scar Massage (when incision is fully healed) CRITERIA FOR PROGRESSION TO PHASE 2

20 no lag SLR Normal gait Crutch/Immobilizer D/C

ROM: no greater than 5º active extension lag, 110º active flexion

*Phase 2:* Early Rehabilitation Phase

(Approximate time frame: weeks 2 to 6)*

Goals

Full ROM

Improve muscle strength

Progress neuromuscular retraining

EXERCISE SUGGESTIONS ROM:

Low load, long duration (assisted prn) Heel slides/wall slides

Heel prop/prone hang (minimize co-contraction / nociceptor response) Bike (rocking-for-range --- riding with low seat height)

Flexibility stretching all major groups

Strengthening:

Quadriceps:Quad sets, Mini-squats/wall-squats, Steps-ups, Knee extension from 90o to 40o, Leg press

Shuttle Press without jumping action Hamstrings:Hamstring curls, Resistive SLR with sports cord Other Musculature:

Hip adduction/abduction: SLR or with equipment

Standing heel raises: progress from double to single leg support

Seated calf press against resistance

Multi-hip machine in all directions with proximal pad placement

Neuromuscular training: Wobble board, Rocker board, Single-leg stance with or without equipment

(e.g. instrumented balance system), Slide board

Cardiopulmonary: Bike, Elliptical trainer, Stairmaster

CRITERIA FOR PROGRESSION TO PHASE 3

Full ROM

Minimal effusion/pain

Functional strength and control in daily activities

IKDC Question # 10 (Global Rating of Function) score of > 7 (See page 7)

*Phase 3:* Strengthening & Control Phase

(Approximate time frame: weeks 7 to 12)

Goals

Maintain full ROM

Running at 12 weeks if without pain or swelling - BEGIN RUNNING ONLY WHEN QUAD STRENGTH 70% OF NON-OP SIDE, SEEN BY MD,

Hopping without pain, swelling or giving-way

EXERCISE SUGGESTIONS

Strengthening: Squats, Leg press, Hamstring curl, Knee extension 90o to 0o, Step-ups/down, Lunges, Shuttle, Sports cord, Wall squats

Neuromuscular Training: Wobble board / rocker board / roller board, Perturbation training, Instrumented testing systems, Varied surfaces

Cardiopulmonary:Straight line running on treadmill or in a protected environment (NO cutting or pivoting) All other cardiopulmonary equipment

CRITERIA FOR PROGRESSION TO PHASE 4

Running without pain or swelling

Hopping without pain or swelling (Bilateral and Unilateral) Neuromuscular and strength training exercises without difficulty

*Phase 4:* Advanced Training Phase

(Approximate time frame: weeks 13 to 16)

Goals:

Running patterns (Figure-8, pivot drills, etc.) at 75% speed without difficulty

Jumping without difficulty

Hop tests at 75% contralateral values (Cincinnati hop tests: single-leg hop for distance, triple-hop for distance, crossover hop for distance, 6-meter timed hop)

EXERCISE SUGGESTIONS

Aggressive Strengthening: Squats, Lunges, Plyometrics Agility Drills: Shuffling, Hopping, Carioca, Vertical jumps Running patterns at 50 to 75% speed

Initial sports specific drill patterns at 50 - 75% effort

Neuromuscular Training:Wobble board / rocker board / roller board, Perturbation training, Instrumented testing systems,Varied surfaces

Cardiopulmonary: Running, Other cardiopulmonary exercises

CRITERIA FOR PROGRESSION TO PHASE 5

Maximum vertical jump without pain or instability

75% of contralateral on hop tests

Run at 75% speed without difficulty

IKDC Question # 10 (Global Rating of Knee Function) score of > 8 (See page 7)

**Phase 5: Return-to-Sport Phase**

**(Approximate time frame: weeks 17 to 26)**

Goals

90% contralateral strength

90% contralateral on hop tests

Sport specific training without pain, swelling or difficulty

EXERCISE SUGGESTIONS Aggressive Strengthening

Squats Lunges Plyometrics

Sport Specific Activities Interval training programs Running patterns in football Sprinting

Change of direction

Pivot and drive in basketball

Kicking in soccer

Spiking in volleyball

Skill / biomechanical analysis with coaches and sports medicine team

Return-To-Sports Evaluation Recommendations: Hop tests (single-leg hop, triple hop, cross-over hop, 6 meter timed-hop) Isokinetic strength test (60°/second)

Vertical jump

Deceleration shuttle test

MOON outcomes measure packet (mandatory; should be completed post-testing)

Return-To-Sports Criteria:

No functional complaints

Confidence when running, cutting, jumping at full speed

85% contralateral values on hop tests
