# Supplementary material for: The relationship between psychological readiness to return to sport and kinesiophobia in teens and young adults after anterior cruciate ligament reconstruction
Source: Front Psychol. 2025 Oct 10;16:1623398. doi: 10.3389/fpsyg.2025.1623398 (PMC12549314; doi:10.3389/fpsyg.2025.1623398)
Supplement: Supplementary file 2 [file Data_Sheet_2.pdf]

# ACL Rehabilitation Guidelines

The following ACL rehabilitation guidelines are based on a review of the randomized controlled trials related to ACL rehabilitation. For many aspects of ACL rehabilitation there are either no studies that qualify as "best-evidence" or the number of studies is too few for conclusions to be drawn with confidence. In these circumstances, the recommendations are based upon the guidance of an expert panel

The guidelines have been developed to service the spectrum of ACL injured people (non-athlete ↔ elite athlete). For this reason, **example exercises** are provided instead of a highly structured rehabilitation program. **Attending rehabilitation specialists should tailor the program to each patient's specific needs**.

The multi-center nature of the group necessitates that the ACL Rehabilitation Program only include treatment methods that can be employed at all sites without purchasing expensive equipm ent. Consequently, some treatment methods with supporting evidence (e.g., using a high-intensity electric stimulation training program for strength, aquatic therapy) are not included in the program because the expert panel believed that it is unreasonable to expect all sites to carry out such treatments.

Progression from one phase to the next is based on the patient demonstrating readiness by achieving **functional criteria rather than the time elapsed since surgery**. The timeframes identified in parenthes es after each Phase are *approximate* times for the average patient, **NOT** guidelines for progression. Some patients will be ready to progress soon er than the timeframe identified, whereas others will take longer.

The *recommended* number of visits to the rehabilitation specialist (including visits merely for evaluation / exercise progression) is **16 to 24** visits with the majority of the visits occurring early (**BIW x 6 weeks**). However, it is recognized that some patient's health plans are severely restrictive. For this reason, the *minimum* number of post-ACL reconstruction visits to a rehabilitation specialist has been set at **6 visits**

# ACL Rehabilitation Guidelines

**Phase 0: Pre-operative Recommendations**

- Normal gait
- AROM 0 to 120 degrees of flexion
- Strength: 20 SLR with no lag
- Minimal effusion
- Patient education on post-operative exercises and need for compliance
- Educated in ambulation with crutches
- Wound care instructions

**PHASE 1: Immediate Post-operative Phase** (Approximate time frame: Surgery to 2 weeks)

**GOALS**

- Full knee extension ROM
- Good quadriceps control (≥ 20 no lag SLR)
- Minimize pain
- Minimize swelling
- Normal gait pattern

**Crutch Use:** WBAT with crutches (beginning the day of surgery)
**Crutch D/C Criteria:** Normal gait pattern
Ability to safely ascend/descend stairs without noteworthy pain or instability (reciprocal stair climbing)

**Knee Immobilizer:** None (Exception: First 24 hours after a femoral nerve block)

**Cryotherapy:** Cold with compression/elevation (e.g. Cryo-cuff, ice with compressive stocking)

- First 24 hours or until acute inflammation is controlled: every hour for 15 minutes
- After acute inflammation is controlled: 3 times a day for 15 minutes
- Crushed ice in the clinic (post-acute stage until D/C)

**EXERCISE SUGGESTIONS**

**ROM**

- *Extension:* Low load, long duration (~5 minutes) stretching (e.g., heel prop, prone hang minimizing co-contraction and nocioceptor response)

- *Flexion:* Wall slides, heel slides, seated assisted knee flexion, bike: rocking-for-range
- Patellar mobilization (medial/lateral mobilization initially followed by superior/inferior direction while monitoring reaction to effusion and ROM)

**Muscle Activation/Strength**

- Quadriceps sets emphasizing vastus lateralis and vastus medialis activation
- SLR emphasizing no lag
- **Electric Stimulation:** *Optional* if unable to perform no lag SLR
- **Discontinue** use when able to perform *20 no lag SLR*
- Double-leg quarter squats
- Standing theraband resisted terminal knee extension (TKE)
- Hamstring sets
- Hamstring curls
- Side-lying hip adduction/abduction (Avoid adduction moment in this phase with concomitant grade II – III MCL injury)
- Quad/ham co-contraction supine
- Prone Hip Extension
- Ankle pumps with theraband
- Heel raises (calf press)

**Cardiopulmonary**

- UBE or similar exercise is recommended

**Scar Massage** (when incision is fully healed)

**CRITERIA FOR PROGRESSION TO PHASE 2**

- 20 no lag SLR
- Normal gait
- Crutch/Immobilizer D/C
- ROM: no greater than 5º active extension lag, 110º active flexion

**PHASE 2: Early Rehabilitation Phase** (Approximate timeframe: weeks 2 to 6)

**GOALS**

- Full ROM
- Improve muscle strength
- Progress neuromuscular retraining

# ACL Rehabilitation Guidelines

## EXERCISE SUGGESTIONS

### ROM

- Low load, long duration (assisted prn)
- Heel slides/wall slides
- Heel prop/prone hang (minimize co-contraction / nociceptor response)
- Bike (rocking-for-range! riding with low seat height)
- Flexibility stretching all major groups

### Strengthening

*Quadriceps:*
- Quad sets
- Mini-squats/wall-squats
- Steps-ups
- Knee extension from 90º to 40º
- Leg press
- Shuttle **Press without jumping action**

*Hamstrings:*
- Hamstring curls
- Resistive SLR with sports cord

*Other Musculature:*
- Hip adduction/abduction: SLR or with equipment
- Standing heel raises: progress from double to single leg support
- Seated calf press against resistance
- **Multi-hip machine in all directions with proximal pad placement**

### Neuromuscular training

- Wobble board
- Rocker board
- Single-leg stance with or without equipment (e.g. instrumented balance system)
- Slide board
- Fitter

### Cardiopulmonary

- Bike
- Elliptical trainer
- Stairmaster

# ACL Rehabilitation Guidelines

**CRITERIA FOR PROGRESSION TO PHASE 3**

- Full ROM
- Minimal effusion/pain
- Functional strength and control in daily activities
- IKDC Question # 10 (Global Rating of Function) score of $\geq$ 7 *(See page 9)*

**PHASE 3: Strengthening & Control Phase** (Approximate timeframe: weeks 7 through 12)

**GOALS**

- Maintain full ROM
- Running without pain or swelling
- Hopping without pain, swelling or giving-way

**EXERCISE SUGGESTIONS**

**Strengthening**

- Squats
- Leg press
- Hamstring curl
- Knee extension 90º to 0º
- Step-ups/down
- Lunges
- Shuttle
- Sports cord
- Wall squats

**Neuromuscular Training**

- Wobble board / rocker board / roller board
- Perturbation training
- Instrumented testing systems
- Varied surfaces

**Cardiopulmonary**

- Straight line running on treadmill or in a protected environment (NO cutting or pivoting)
- All other cardiopulmonary equipment

**CRITERIA FOR PROGRESSION TO PHASE 4**

- Running without pain or swelling

- Hopping without pain or swelling (Bilateral and Unilateral)
- Neuromuscular and strength training exercises without difficulty

**PHASE 4: Advanced Training Phase** (Approximate timeframe: weeks 13 to 16)

**GOALS**

- Running patterns (Figure-8, pivot drills, etc.) at 75% speed without difficulty
- Jumping without difficulty
- Hop tests at 75% contralateral values (Cincinnati hop tests: single-leg hop for distance, triple-hop for distance, crossover hop for distance, 6-meter timed hop)

**EXERCISE SUGGESTIONS**

**Aggressive Strengthening**

- Squats
- Lunges
- Plyometrics

**Agility Drills**

- Shuffling
- Hopping
- Carioca
- Vertical jumps
- Running patterns at 50 to 75% speed (e.g. Figure-8)
- Initial sports specific drill patterns at 50 – 75% effort

**Neuromuscular Training**

- Wobble board / rocker board / roller board
- Perturbation training
- Instrumented testing systems

- Varied surfaces

**Cardiopulmonary**

- Running
- Other cardiopulmonary exercises

**CRITERIA FOR PROGRESSION TO PHASE 5**

- Maximum vertical jump without pain or instability
- 75% of contralateral on hop tests
- Figure-8 run at 75% speed without difficulty
- IKDC Question # 10 (Global Rating of Knee Function) score of $\geq$ 8 *(See page 9)*

**PHASE 5: Return-to-Sport Phase** (Approximate timeframe: weeks 17 to 20)

**GOALS**

- 85% contralateral strength
- 85% contralateral on hop tests
- Sport specific training without pain, swelling or difficulty

**EXERCISE SUGGESTIONS**

**Aggressive Strengthening**

- Squats
- Lunges
- Plyometrics

**Sport Specific Activities**

- Interval training programs
- Running patterns in football
- Sprinting
- Change of direction
- Pivot and drive in basketball
- Kicking in soccer
- Spiking in volleyball
- Skill / biomechanical analysis with coaches and sports medicine team

**RETURN-TO-SPORT EVALUATION RECOMMENDATIONS:**

- Hop tests (single-leg hop, triple hop, cross-over hop, 6 meter timed-hop)

- Isokinetic strength test (60°/second)
- Vertical jump
- Deceleration shuttle test
- MOON outcomes measure packet (mandatory; should be completed post-testing)

**RETURN-TO-SPORT CRITERIA:**

- No functional complaints
- Confidence when running, cutting, jumping at full speed
- 85% contralateral values on hop tests
- IKDC Question # 10 (Global Rating of Knee Function) of $\geq$ 9 *(See page 9)*

**IKDC Question #10**

How would you rate the function of your knee on a scale of 0 to 10 with 10 being normal, excellent function and 0 being the inability to perform any of your usual daily activities which may include sports?

CURRENT FUNCTION OF YOUR KNEE:

| Cannot perform Daily activities | | | | | | | | | | No Limitation |
|---|---|---|---|---|---|---|---|---|---|---|
| 0 | 1 | 2 | 3 | 4 | 5 | 6 | 7 | 8 | 9 | 10 |
| ☐ | ☐ | ☐ | ☐ | ☐ | ☐ | ☐ | ☐ | ☐ | ☐ | ☐ |

# ACL RECONSTRUCTION REHABILITATION PROTOCOL

## PHASE I: IMMEDIATE POST-OPERATIVE PHASE

**Goals:** Control inflammation/effusion
Restore full terminal knee extension
Gradually improve knee flexion
Re-establish quad control and ability to perform SLR

**Post-op Days 1-14**

- Remove bulky dressings on 3rd day, leave steri-strips intact
- Brace locked in full extension
    - Ok to unlock or remove brace 4-5 times/day for ROM exercises
    - Sleep with brace locked in extension to discourage flexion contracture at night time
- TTWB/PWB with crutches until able to walk without a limp
- Exercises
    - Work on gentle self-ROM
    - Ankle pumps
    - Quad sets
    - Short arc quads (0-30 degrees terminal knee extension)
    - Straight leg raise
- Muscle stimulation (NMES Unit) to quads if available
- Ice Man 20 minutes at a time, several times per day to control pain and post-operative swelling
- Elevate leg above heart as much as possible

## PHASE II: EARLY REHABILITATION PHASE

**Goals:** Control inflammation, minimal joint effusion
Quad control
Full knee ROM
Diminish pain

**Weeks 3-4 Post-op**

- Brace may be discontinued after patient is able to perform SLR
- Discontinue use of crutches when able to walk without a limp
- Progress full, pain-free knee ROM and joint mobilizations (especially inferior guided patellofemoral joint mobilizations to restore flexion)
- Exercises:
    - Continue exercises from Phase I as needed
    - Stationary bike at low resistance (progressing gradually)

- Forward, lateral and retro step ups (4”-6”)
- Heel raise progression
- Hamstring curls (low weight, high reps in pain free ROM)
- Early balance and proprioception
- Mini-squats (progress to single leg when quad control is sufficient)

**** DO NOT utilize knee extension machines or perform decline bench core exercises that lock the lower extremity into the unit, as this places abnormal strain on the ACL ****

- Muscle stimulation (NMES Unit) or BFR to quads if available
- IceMan as needed

**PHASE III: PROGRESSIVE STRENGTHENING/NEUROMUSCULAR CONTROL PHASE**

**Goals:** Improve quad strength and control
Normalize gait
Minimal to no joint effusion
Pain-free

**<u>Weeks 5-6</u>**

- Exercises
  - Restore residual ROM deficits
  - Quadriceps/VMO strengthening exercises
  - Stair master, rowing, Nordic track (5 min. and progress as tolerated)
  - Mini-squats
  - Gait training
  - Controlled leg press
  - Hip and core strengthening
  - Progress proprioception (progress to single leg, introduce foam cushion or rocker board, perturbations, eyes open vs. eyes closed, etc.)
  - Phase I Aquatic Therapy (Only after the incision site is completely closed and sloughing) - Pool walking forward and lateral, bicycling/high knees, flutter kicks with knee remaining extended, ½ squats, weightless jogging
- Continue NMES and/or BFR if available
- Upper body and core workouts as desired

**<u>Weeks 7-12</u>**

- Exercises
  - Progress quad, hamstring, hip and core strengthening (leg press, single leg RDL’s, step up progression, utilization of supersets)
  - Controlled step downs emphasizing good mechanics with knee tracking
  - Progress balance and proprioception (BAPS, single leg balance progression, etc.)
  - Can introduce jogging at 8 weeks if patient demonstrates appropriate mechanics and good muscular endurance (50-75% pace on level surfaces

in a straight line, progressing gradually. No cutting, twisting, pivoting, uneven surfaces or contact sports activity)

- Continue BFR if available
- Phase II Aquatic Therapy (treading water, kickboard swimming, pool running, shuffling and carioca, swimming, modified aquatic sports)

**PHASE IV: ADVANCED STRENGTHENING/FUNCTIONAL TRAINING**

**Goals:** Normalize lower extremity strength
Normalize neuromuscular control and proprioception

**Weeks 13-14**

- Progress controlled strengthening exercises
- Advance running progression - can introduce jogging outdoors on flat, even surfaces
- Introduce controlled deceleration training and drills
- Begin controlled lateral movements
- Progress jumping and hopping

**Weeks 15-20**

- Advance running progression
- Advance lateral movements
- Progress jumping and hopping
- Introduce sport-specific drills
- Complete first round of functional testing with Research Lab at 4 months (includes single leg squatting, retro step-up, single leg forward hop, single leg timed hop, crossover triple hop)

**Weeks 21-24 (or clearance)**

- Fine-tune activities
    - Cutting/pivoting
    - Jumping/hopping
    - Strengthening
    - Non-contact sports activities
- Normalize endurance
- Functional/strength testing all ≥ 90% contralateral side
- Measured for functional brace if needed
- Complete functional testing with therapist orResearch Lab at 6 months
- See surgeon as scheduled for final clearance

# Anterior Cruciate Ligament Reconstruction

## Post-Operative Program (ver 1.0)

### *Phase I: Post – Op to Week 2*

-Wound Care/Dressing Change
-Reduce Swelling & Pain Control
-Achieve & Maintain **FULL KNEE EXTENSION**
-WBAT with brace locked at 0˚; once SLR x10 w/o lag → wean from crutches/brace and normalize gait
-Patellar mobilization
-Utilization of NMES for Quadriceps activation
-Focus on Hamstring/Quadriceps Co-contraction (**Unless Hamstring Graft Used – In that case; no active Hamstring contraction for 8 weeks post-op)*
-Ensure available ankle DF and hip mobility is adequate

***If full extension is NOT achieved by week 2; increase frequency of treatment with that being primary focus****

### *Phase II: Week 2 to Week 8:* Requires Full Knee Ext & SLR x10 w/o Lag

-Continue with treatment as prior PRN for reinforcement
-Progress ROM to 90˚ of flexion; avoid aggressive overpressure; then allow flexion to increase naturally
-Achieve SLR **WITHOUT** any lag – even a small amount of flexion will stress graft
-Closed Kinetic Chain (CKC) activity with focus of proprioception/balance (Initiate activity done in Bilateral positions and transition to unilateral as stability improves)

- -Utilize foam, trampoline, BOSU, etc. for perturbation forces.
- -Hip strength (Bridges, Clams, Hip Series, etc.)
- -Leg press, Lunge, side-lunge (Mindful of avoidance of Hip IR/Genu Valgum deviations, work in 90°-30° of flexion early, do not let knees move anterior to toes)
- -Squatting done with heels on floor and 30°-45° forward tilt of trunk to engage Hamstrings

### *Phase III: Week 8 to Week 12 "The Cool-Down"*

-Emphasis on pt. education leading up to this point – Pt needs to the know "the plan" going forward
-**IF** adequate Quadriceps/Hamstring Strength and Full ROM of knee are achieved; Reduce frequency of visits to 1x/week.

- -Graft is at weakest point & reduced frequency may improve retention of patient over duration of rehab process.

### *Phase IV: Week 12 to Week 16*

-Y-Balance Testing (Establish baseline scores)
-Progress intensity and volume of functional training
-Increase the amplitude of ROM utilized with CKC activity with appropriate technique maintained.
-Initiate forward jogging

### *Phase V: Week 16 to Week 24*

-Hop testing
-Repeat Y-Balance until ≤4 cm difference to unaffected side
- Cutting & agility training If achieves ≥ 85% of contralateral limb with 1RM and hop testing initiate
-Focus on sport-specific/work hardening activity and compound movement training

### *Phase VI: Week 24+*

-Focus on Power & Plyometrics always emphasizing form/technique
-Repeat Hop testing until ≥ 95% of unaffected side
-Supervised return to controlled sport activity

## ACL / PCL REHAB MODIFICATIONS

☐ **MENISCAL REPAIR** Z98.890

- ☐ **(ALL INSIDE) (INSIDE OUT) (MEDIAL) (LATERAL)**
  - WBAT with knee locked in extension x 6 weeks then wean from Brace
  - No Limit to OKC ROM
  - No WB on bent knee x 4 weeks, then CKC activities 0°-60°
  - After 4 weeks CKC not past 90°
- ☐ **(ROOT) (RADIAL) (TRANSPLANT) (MEDIAL) (LATERAL)**
  - NWB Weeks 1-2 in Bledsoe Brace locked @ 0° in Full Extension.
  - TTWB Weeks 2-4 in Bledsoe Brace locked @ 0° in Full Extension
  - PWB 50% Weeks 5-6 in Bledsoe Brace locked @ 0° in Full Extension
  - WBAT and wean from brace after 6 weeks
  - Limit A/P ROM
    from 0° to 90° for 0-4 weeks, then 0° to 120° for 4-6 weeks
  - After 6 weeks no OKC ROM Limitations, CKC not past 90°

☐ **LCL / PLC**. S83.429D

Protect against Varus / Posterolateral laxity—Minimize Varus Stresses

- Post-op brace locked in extension NWB x 2 weeks
- 50% PWB with knee locked in extension 2-4 weeks
- WBAT with knee locked in extension 4-6 weeks- wean from crutches
- ROM 0-90 degrees or per PCL guidelines – AVOID hyperextension
- Perform flexion passively to avoid undue stress imposed by active contraction of hamstrings x 8 weeks
- Progress OKC exercises as tolerated by PF joint (limit 60 degrees if with PCL reconstruction also)
- CKC exercises as tolerated at 6 weeks and FWB
- Avoid: OKC Flexion, Hip abduction with resistance distal to knee

☐ **MCL.** S83.419D

**Week 0-4**

- NWB Weeks 1-2, TTWB Weeks 2-4 in Bledsoe Brace locked @ 0° in Full Extension.
- ROM 0-90
- Avoid Valgus stress to knee ( No Hip adduction leg lifts)

**Week 4-8**

- WBAT
- NO ROM Limitations
- Wean from Brace after 6 weeks

☐ **BEAR Implant**

- Defer to official BEAR Rehab Protocol
- 50% PWB with knee locked in extension 0-4 weeks
- WBAT with knee locked in extension 4-6 weeks- wean from crutches
- ROM: 0-45 weeks 0-2, 0-90 weeks 2-4
- Week 7 wean from brace and ROM 0-110
- Week 10 no ROM restrictions, progress as per standard ACL protocol

# ACL QUADRICEPS TENDON AUTOGRAFT RECONSTRUCTION GUIDELINES

**General notes:**
"As tolerated" should be understood to include with safety for the reconstruction/repair; pain, limp, swelling, or other undesirable factors are indicators that you are doing too much too soon. If any of these should occur, decrease activity level, ice and elevate the leg.
Apply ice to the knee for 15-20 minutes following each exercise, therapy, or training session. Return to sport based on provider team (physician, physician assistant, athletic trainer, therapist) input and appropriate testing.
All times and exercises are to serve as guidelines. Progression through the protocol should be based upon criteria as opposed to dates listed and will vary depending on each individual patient. Progress will be agreed upon by the patient and his/her team of providers.

**PHASE I (Weeks 1-4)**
GENERAL GUIDELINES
• Focus on protection of graft during primary revascularization (8 weeks) and graft fixation (4-6 weeks)
• No bathing/swimming until after suture removal and wounds healed
• Showering permitted with water-proof covering over sutures (Tegaderm/OpSite)
  • Driving: Must be off all narcotic pain meds when operating vehicle
    o 1 week for automatic cars, left leg surgery
    o 2-4 weeks for standard/manual cars or right leg surgery
• Crutches for ambulation for 1-4 weeks as determined by MD/PT. Discontinue when gait is normalized (i.e. – no limp present)
  • Brace use:
    o WBAT locked in extension for 1 week per MD/PT
    o Sleep with brace locked in extension for 1 week or per MD/PT order
• Return to work as determined by MD/PT dependent on work demands
• Use ice and elevation for swelling/pain control
• If performed in conjunction with meniscal repair or other surgery, defer to most restrictive guidelines.

GOALS
• Protect repaired structures
• Educate patient on rehab progression
• Decrease inflammation and swelling
• Control pain
• Symmetrical active and passive knee extension/hyperextension ROM. Avoid hyperextension greater than 10 degrees
• Passive knee flexion to 90 degrees
• Restore normalized gait on level surfaces within precautions
• Restore full patellar mobility
• Demonstrate good quad activation

EXERCISES
• Quad sets
• Patellar mobilizations

• Heel slides, AAROM prone knee flexion, seated flexion stretch
• Passive knee extension
• Hip SLR in 4 planes (in brace until can perform without quad lag)
• Hamstring and gastrocnemius stretch, non-weightbearing
• Aquatic therapy after sutures removed and wounds completely healed
• UBE (arm bike)
• Stationary bike for ROM/strength (starting POD#14)
• Closed kinetic chain quad strengthening (wall sits, step-ups, mini-squats, leg press)
• Initiate proprioceptive exercises (single leg balance, ball toss, balance beam, BOSU, Airex)
• Calf strengthening

MODALITIES
• NMES (neuromuscular electrical stimulation for quadriceps atrophy, strengthening as needed)
• HVPC (high volt pulsed current) for effusion (swelling) reduction, as needed
• Cryotherapy 6-8 times/day for 15-20 minutes each time

CRITERIA TO ADVANCE TO PHASE II
• Knee ROM: 0-90 degrees, AKHE
• Perform SLR without quad lag
• Normalized gait per precautions
• Normal patellar mobility
• Minimal swelling/inflammation

**PHASE II (Weeks 4-12)**

*Do NOT initiate new activities during post-op week 8 due to graft vulnerability*

GOALS
• Eliminate inflammation and swelling
• Full knee ROM (0-135 degrees)
• Active knee hyperextension
• Normal gait on all surfaces without brace or assistive device
• Improve lower extremity strength
• Demonstrate stability with dynamic knee activities (no varus/valgus deviations)

EXERCISES
• Advance ROM
• Progress hip, quad, hamstring, calf strengthening. Avoid open chain quad strengthening 30-0 degrees
• Cross-training machines for conditioning
• Advance lower extremity flexibility
• Advanced aquatic exercises

CRITERIA TO ADVANCE TO PHASE III
• Full knee ROM, including AKHE
• Demonstrates good quad strength with exercises
• Normal gait on all surfaces at community level distances
• Minimal swelling/inflammation
• No pain with exercises

**PHASE III (Weeks 12-24)**

GOALS
• Increase strength to >85% non-involved extremity
• Advance proprioception exercises
• Improve aerobic endurance
• Initiate plyometric exercises
• Physician clearance to initiate return to running and functional progression
**WILL NEED FUNCTIONAL TESTING AT 4 MONTHS POST-OP**

EXERCISES
• Spin bike
• Cybex training
• Pre-running exercises (low skips, punch steps, double punch steps, hurdle walks, high skips, kickbacks, step-overs)
• Advance proprioceptive exercises (BOSU, single leg dynamic balance, dual task balance)
• Agility drills (ladder, side shuffles, crossovers, backwards run, quick start/stops, zig-zags, cutting)
• Jump training (shuttle training, trampoline, landing technique, box jumps, single leg hops, tuck jumps)
• Return to running – treadmill, with transition to level outdoor surfaces
• Continue strengthening - advance resistance and repetitions (ball hamstring curls, single leg press, core stabilization)

CRITERIA TO ADVANCE TO PHASE IV
• Lower extremity strength greater than or equal to 85% of non-involved by Cybex test
• Single leg hop test greater or equal to 85% of non-involved
• No pain with forward running, agilities, jump training, or strengthening
• Good knee control with single leg dynamic proprioceptive activities
**WILL NEED FUNCTIONAL TESTING AT 6 MONTHS POST-OP**

**PHASE IV (Weeks 24-full return)**

GOALS
• Full return to sport activity
• Equal bilateral lower extremity strength
• Equal bilateral balance, proprioception, power in lower extremity
• 100% global function rating
**WILL NEED FUNCTIONAL TESTING AT 9 MONTHS POST-OP**
• Advance above exercises
• Gradually increase level of participation in sports-specific activities
• Running on all surfaces
